# Surgery

**Published:** 1898-10

**Authors:** John Parmenter, Nelson W. Wilson

**Affiliations:** Buffalo, N. Y., Professor of anatomy and adjunct professor of clinical surgery, Buffalo University Medical College; Buffalo, N. Y.


### SURGERY.

Conducted by JOHN PARMENTER, M. D., Buffalo, N. Y.,
Professor of anatomy and adjunct professor of clinical surgery, Buffalo University Medical College,

AND

NELSON W. WILSON, M. D., Buffalo, N. Y.

### TRANSPLANTATION OF THE RECTUS MUSCLE IN INGUINAL HERNIA.

JOSEPH C. BLOODGOOD (*Johns Hopkins Hospital Bulletin*) gives a preliminary report on "transplantation of the rectus muscle in certain cases of inguinal hernia, in which the conjoined tendon is obliterated." He divides inguinal hernia into two groups: one of which the conjoined tendon is wide and firm and one in which it is practically obliterated.

The procedure is the typical Halstead operation up to the point of inserting the deep sutures. Before this is done the sheath of the rectus muscle is exposed and divided from the insertion of the muscle upward for 5 c.m. The outer border of the belly of the muscle is caught with two or three sutures of heavy black silk which, used as retractors, pull the muscle downward and outward. Then the deep sutures of silver wire are inserted as in Halstead's operation, with the addition that the four sutures below the transplanted cord include the sheath of the rectus and the muscle itself. When these sutures are tied the rectus is approximated to Poupart's ligament and the aponeurosis of the external oblique from a position just below the transplanted cord down to the symphysis pubis, in addition to the divided and transplanted internal oblique muscle.

The term obliterated, as applied to the conjoined tendon, is used because the extreme condition is more likely to be an acquired one than congenital. The conjoined tendon may be congenitally very narrow or attenuated. The important point to be recognised is that the conjoined tendon is either very narrow or very attenuated or obliterated and that Hasselbach's triangle has lost its strongest support, the conjoined tendon, and that something must be transplanted to act as a substitute for the defect; this something is the rectus muscle.

---

### A DRY DRESSING.

Acetanilid 48 parts, boric acid 15 parts, finely powdered starch 35 parts and carbolic acid 2 parts is reported as having given excellent results as a dry dressing for granulating surfaces and sutured wounds.

---

### A PLASTER FOR BURNS.

Vergely, of Bordeaux, in burns of the first and second degree, covers the part with a thick paste of calcined magnesia and water and lets it dry on. Pain usually ceases at once and healing progresses rapidly as a rule. He also uses potassium nitrate as a bath, in the proportion of a few teaspoonfuls to the basin of water. If pain returns after immersion of the part, add a little more potassium. Compresses wet with the solution in a measure take the place of a bath.

---

### TO PREVENT NAUSEA AFTER ANESTHESIA.

Capt. E. B. Frick, assistant surgeon, U. S. A., at Presidio, has found that inhalation of acetum aromaticum prevents nausea after anesthesia. Inhalations may be continued an hour.

---

### TO STOP NOSE-BLEED.

Dr. A. C. Smith (*Clinique*) gives this routine for checking nose bleed. Grasp the nose between the thumb and forefinger and press backward against the alveolar border of the maxilla and downward against the teeth. "This compresses the lateralis nasi and septal arteries." He reports satisfactory results following the use of tannin and acetanilid.

---

### THE CAUSE OF DEATH AFTER BURNS.

Ageleo and Parascandalo gives the cause of death after burns as a toxic ptomaine, not, however, due to the toxins produced in the burns by bacteria or to anatomic changes which the corpuscles or organs of the burned area may have undergone.

### TREATMENT OF SINGLE PROLAPSE OF THE RECTUM.

Dr. Rehn, of Frankfort, treats simple, recurring prolapse of the rectum by repeated momentary cauterisation of the anal mucous membrane at intervals of five days with lunar caustic. A cure usually results after five to eight applications. The cauterisation should be superficial and results in contraction of the sphincter.

---

### OSTEOMYELITIS FOLLOWING TRAUMATISM.

K. Geronne (*Centrablatt für Kinderheilk.*) reports a case of osteomyelitis following trauma. A boy playing football was struck on the foot by the ball and fell striking his left hand. The foot and hand became painful the following day; eight days later the diagnosis of osteomyelitis was made; operative measures were resorted to, but one bone after another became involved and death resulted two months after the accident. *Pediatrics* in summing up says, in giving possible causes of osteomyelitis after slight injuries: Excitors of pus, by chance present in the blood, may escape from the latter by means of torn vessels and crushed portions of bone; cocci taking advantage of a favorable point for colonising, caused by the purulent inflammation of the soft tissues, may pass into the circulation; the patient may have had a previous attack of osteomyelitis, and the bacteria being still present encapsulated in the bone, are stirred into action by a new traumatism. It may be that slight abrasions are present, for example, on the foot, and these may become the point of entrance for schizomycetes. This latter condition may possibly be one of great importance and thus account for the frequency with which children are attacked by osteomyelitis, they being more exposed than adults to these germs and to slight traumatisms.

---

### THE LATER FORM OF HEREDITARY SYPHILIS.

V. N. Alexejew, of Moscow, reports a case of the later form of hereditary syphilis and its treatment. Child, 12 years old, well nourished and developed, mucous membranes pale; slight swelling of cervical lymphatics; liver markedly enlarged, hard, felt in mamillary line, 6 c.m. below the edge of the ribs; surface of right lobe hobnailed; two movable tumors on left lobe; liver sensitive to pressure; spleen enlarged. For eleven days the child was put on 0.9 of potassium iodide, daily; the following thirty-five days the dose was increased to 1.5 daily. Two months from the beginning of the treatment the child was discharged cured.

---

Dr. J. T. Rugh (*Polyclinic*) suggests the use of sugar to facilitate the removal of plaster from the hands.

---

Dr. J. C. Oliver (*Cincinnati Lancet Clinic*) reports a case of colloid carcinoma of the cecum. The entire cecum, three inches of small intestines and an inch and a half of the large intestine were removed. The divided ends of the large and small intestines were connected by a Murphy button. Recovery was rapid and uninterrupted. The appendix was not included in the carcinomatous process.

---

There were seventy-one papers on surgery read before the American Medical Association at the Denver meeting.

---

The *Indiana Medical Journal* says plaster sets much more efficiently with potassium sulphate than with sodium chloride.

---

Emory Lanphear gives the following as surgical sins: (1) Operating in hopeless cases; (2) Delaying opinion as to the gravity of a disease; (3) Failure to operate in depressed fracture of the skull; (4) Pretending to be clean; (5) Undercharging in order to secure an operation; (6) Stealing patients; (7) representing capital operations as trifling; (8) Keeping patients too long under chloroform *ariston metron*. Unwise speed is bad; chronic surgery is worse.

---

And now Dr. Murphy's method of treating phthisis by injecting nitrogen into the pleura has been attacked as "not new." Dr. W. H. Meyer, of Fort Wayne, Ind., who has written a letter to a newspaper, makes the attack. He quotes from Dr. Paget's recent work, and states that in Rome in 1894, Prof. C. Farlanini reported cases treated by forming an artificial pneumothorax and states that "the pleura bears air well, but that nitrogen is preferable, as it is absorbed more slowly." The method is described in Volume III. proceedings of the *International Medical Congress*, 1894. Similar views was advanced in 1891 by Hilton Flagg, and in 1881, Dr. Herard, Hotel Dieu, Paris, discovered that an accidental hydro-pneumothorax arrested phthisis and "became an unhoped for means of safety for the patient."

